# Printed Two-Dimensional Materials for Flexible Photodetectors: Materials, Processes, and Applications

**DOI:** 10.3390/s25041042

**Published:** 2025-02-10

**Authors:** Lingxian Kong, Shijie Wang, Qi Su, Zhiyong Liu, Guanglan Liao, Bo Sun, Tielin Shi

**Affiliations:** 1 State Key Laboratory of Intelligent Manufacturing Equipment and Technology, Huazhong University of Science and Technology, Wuhan 430074, China; lingxiankong@hust.edu.cn (L.K.); zhiyong_liu@hust.edu.cn (Z.L.); guanglan.liao@hust.edu.cn (G.L.); 2 School of Aerospace Engineering, Huazhong University of Science and Technology, Wuhan 430074, China; shijiewang@hust.edu.cn (S.W.); qisu@hust.edu.cn (Q.S.); 3 Shenzhen Research Institute, Huazhong University of Science and Technology, Shenzhen 518057, China

**Keywords:** 2D materials, solution processing, printing technologies, flexible photodetectors

## Abstract

With the rapid development of micro-nano technology and wearable devices, flexible photodetectors (PDs) have drawn widespread interest in areas such as healthcare, consumer electronics, and intelligence interfaces. Two-dimensional (2D) materials with layered structures have excellent optoelectronic properties and mechanical flexibility, which attract a great deal of attention in flexible applications. Although photodetectors based on mechanically exfoliated 2D materials have demonstrated superior performance compared to traditional Si-based PDs, large-scale manufacturing and flexible integration remain significant challenges for achieving industrial production. The emerging various printing technology provides a low-cost and highly effective method for integrated manufacturing. In this review, we comprehensively introduce the most recent progress on printed flexible 2D material PDs. We first reviewed the most recent research on flexible photodetectors, in which the discussion is focused on substrate materials, functional materials, and performance figures of merits. Furthermore, the solution processing for 2D materials coupled with printing functional film strategies to produce PDs are summarized. Subsequently, the various applications of flexible PDs, such as image sensors, healthcare, and wearable electronics, are also summarized. Finally, we point out the potential challenges of the printed flexible 2D material PDs and expect this work to inspire the development of flexible PDs and promote the mass manufacturing process.

## 1. Introduction

Photodetectors (PDs), one of the most widely used sensors, refer to devices that can absorb photons and generate detectable electron–hole pairs, which have many novel applications in fields such as night vision, radiation detection, optical communication, and healthcare [1,2,3,4]. With the rapid development of consumer electronics, the Internet of Things (IoT), and the emergence of the post-Moore’s Law period, the demand for high-performance PDs is increasingly significant, and the research on high-performance PDs is becoming increasingly in-depth. Traditional Si-based optoelectronic devices integrated on rigid substrates indeed exhibit limitations in terms of low light absorption coefficient and lacking mechanical flexibility [5,6]. Flexible PDs with the ability of bendability, foldability, and stretchability have many advantages in wearable devices compared with traditional PDs, especially in terms of wearable healthcare, wearable retinal prostheses, and flexible cameras. Meanwhile, flexible functional materials with superior performance compared to Si-based materials are urgently required, such as good machinal flexibility and durable stability under repeated bending.

In the past decade, 2D materials with atomic thickness have attracted significant attention due to their excellent optoelectronic properties and physical properties, such as high carrier mobility, tunable bandgap, strong light absorptivity, and machinal flexibility [7,8,9]. The dangling bond-free interface between layers of 2D materials eliminates the constraint of lattice mismatch, which makes them easily integrable on the surface of various flexible substrates. Benefiting from the layered structure of 2D materials, as early as 2004, K. S. Novoselov et al. exfoliated monolayer graphene from bulk graphite for the first time [10]. Since then, mechanical exfoliation has been the primary method of accessing 2D materials flakes. The flakes obtained by machinal exfoliation possess high crystallinity, which is still widely used in the research of 2D materials. However, the drawbacks, such as low production yield, uncontrollable thickness, and poor scalability, confine mechanical exfoliation to experimental research [11,12,13]. Industrialization of 2D materials still faces many fundamental challenges, among which industry-controllable production is the most prominent issue. Chemical vapor deposition (CVD) is considered the most promising method for synthesizing large-area 2D materials film [14]. However, the film synthesized by CVD exhibits numerous defects, which impede the industry application of CVD. Additionally, the growth of 2D materials through CVD requires high temperatures ranging from 600 to 900 °C, making it unsuitable for flexible substrates that cannot withstand such heat [1,14]. Consequently, the incompatibility between CVD and flexible substrate inevitably needs additional transfer of the film, which also introduces further defects to the film.

The native layered structure of 2D materials facilitates the solution-processing of exfoliating nanosheets into functional ink. The atomic thickness nanosheets of 2D materials can be scalably assembled into continuous thin-film networks by various printing technologies, such as spraying [15,16,17,18,19], screen printing [7,17,20,21], inkjet printing [22,23,24,25,26,27,28], electrohydrodynamic jet printing [29,30,31,32], and aerosol jet printing [33,34]. In addition, printing technologies based on ink made from 2D material nanosheets can easily deposit films of different thicknesses on flexible substrates. The coupling of 2D material ink printing with flexible substrates is a promising revolution towards low-cost, highly effective, large-scale manufacturing of flexible 2D material PDs. So far, several review articles have discussed the solution-processing of 2D materials and printed electronic devices. However, the discussion mainly puts emphasis on the process of printing technology and ink preparation and reports about the large-scale manufacture of flexible 2D material PDs via printing technology are scarce.

**Scheme 1:**
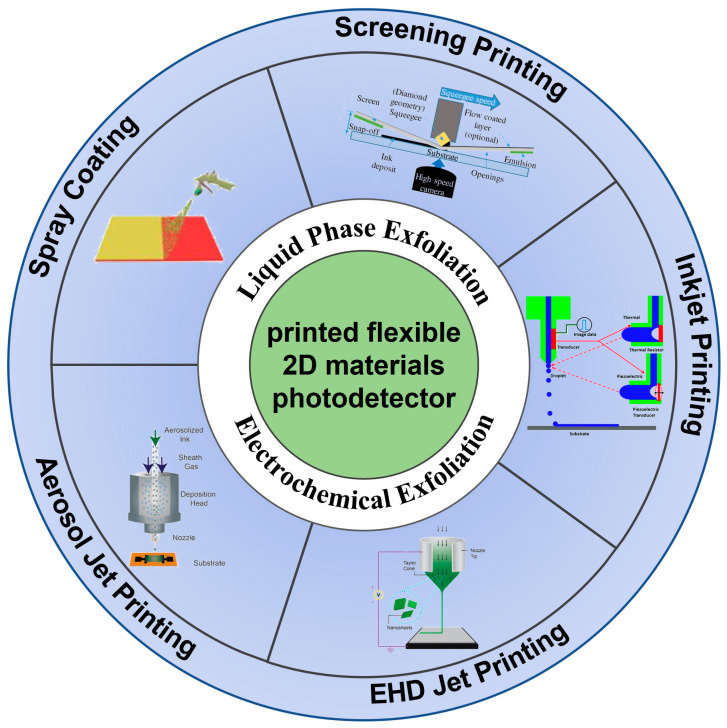
Schematic of flexible 2D material PDs made of printing techniques: spray coating [15], screening printing [20], inkjet printing [28], EHD jet printing [31], and aerosol jet printing [33].

Therefore, this review comprehensively discussed the most recent progress on printed 2D materials flexible photodetectors. Firstly, the typical flexible substrates, 2D materials, and key figures of merit of flexible photodetectors are introduced and discussed, which will help us understand and evaluate the current state of flexible PDs. Then, we present the state of the art on printed flexible 2D material PDs, the dispersed nanosheets ink synthesis method, and various printing technologies, as shown in Scheme 1. Subsequently, we explore the application prospects of printed flexible 2D material PDs. Finally, a conclusion on potential challenges and future expectations in the printed flexible 2D material PDs field is presented.

## 2. Substrate, Functional Materials, and Performance Figures of Merit for Flexible Photodetectors

### 2.1. Substrate Materials

Flexible substrate and functional materials are all core components of flexible photodetectors. The flexibility of PDs is decided by the components of devices, including substrates, functional materials, and the interfacial match between adjacent layers. Polymer films such as polyimide (PI), polyethylene terephthalate (PET), and polyethylene naphthalate (PEN) are commonly utilized as substrates for flexible PDs [24,35,36,37,38]. The flattened surface of polymer films provides a decent adhesion between 2D materials and substrates, which ensures that the active materials will not detach from the substrates under bending, thus providing excellent mechanical stability for a flexible photodetector. Polymer-based flexible substrates have the following benefits: (1) high mechanical strength and chemical stability; (2) flexible and optical clarity; (3) economical fabrication and controlled large-scale production. Mark C. Hersam et al. proposed inkjet-printed solution-processed MoS_2_ nanosheets and graphene nanosheets photodetectors on flexible polyimide substrates [24]. After 500 bending cycles, the device can still maintain invariant sensitivity (I_ph_/I_dark_) and functionality. Subsequently, Sun et al. presented a flexible monolayer MoS_2_ photodetector enhanced by Ag nanocubes localized surface plasmon on PET film [39]. The photoresponse of the flexible PET device remains at 71% of the initial value after bending 10,000 cycles, and the decline mainly occurs during the first 3000 times, indicating the good mechanical endurance and electrical reliability of flexible photodetector based on PET film. In addition, Kong et al. reported an inkjet-printed flexible photoimaging array based on PET substrate; the substrates can withstand 3000 bending cycles while the photocurrent ratio of the device remains above 80% [25]. Despite the numerous advantages of polymer substrates, the poor breathability is a significant problem in wearable electronic applications that involve direct connection with the skin.

Apart from polymer materials, paper, textile, and hydrogel with features like a light weight, nontoxicity, bio-degradability, and breathability are also emerging alternatives for substrates of flexible photodetectors [19,27,40,41,42]. From the cost-effective and environmentally friendly aspects, they are also competitive with traditional polymer materials. For instance, C K Sumesh et al. presented a paper-based flexible PD by simply rubbing WSe_2_ nanosheets on a bio-degradable paper substrate [41]. The photodetector can work in different curvatures, and the total process is eco-friendly and cost-effective. Pratik M. Pataniya et al. designed a green synthesized high-performance WS_2_/graphene heterojunction on a paper substrate [40]. The porous cellulose paper-based PD exhibits stable photoresponse after bending cycles up to 500 and shows piezoresistive photoresponse under inward and outward bending. A C Ferrari et al. utilized polyester fabric and SLG/BP as flexible substrate and channel materials to fabricate flexible PDs [27]. The relative photoresponse (I_ph_ rest/I_ph_ bend) changes about 17% with the radius of the textile ranging from flat to 25 mm, and the photoresponse can retain 82% after 30 cycles. Hydrogel also holds tremendous attention in flexible applications because of its excellent biocompatibility and biodegradability, which make it highly promising in fields related to biomedicine. Qiu et al. mixed black phosphorus with agarose hydrogel to obtain a light-responsive hydrogel drug used for the clinical treatment of cancer [42]. It is important to note that the hydrogel can be degraded and excreted through urine under near-infrared light irradiation and is harmless to humans. The selection of a suitable substrate is crucial for the preparation of flexible photodetectors. The requirements for the flexible substrate vary depending on the terminal device and application environment of the flexible photodetector. For example, PI has better machinal stability and thermal stability, making it favored in high temperature environments such as aerospace electronics. While the PET substrate has higher transparency, therefore, it is used more in scenes that need high transmittance. Although textile is not as stable and flat as polymer materials, its good flexibility and comfort make it play an important role in wearable electronics.

### 2.2. Functional Materials

#### 2.2.1. Graphene and Graphene-like Materials

As the earliest researched 2D material, graphene possesses an extremely narrow bandgap, exceptionally high carrier mobility, and strong light-matter interaction, which make it suitable to be employed as an electrode material and sensitive material in PDs [43]. The nearly zero bandgap in graphene confers it with semi-metallic properties. Its photoresponse extends from visible light to the terahertz region, providing PDs with an exceptionally broad spectral detection capability [27,44]. Compared to silicon, the carrier mobility of graphene is tenfold higher (~20,000 cm^2^V^−1^s^−1^), which makes graphene more competitive in high-frequency photonics applications [45]. Woo Lee et al. fabricated micropatterned graphene electrodes on PI substrates by inkjet printing graphene nanosheets [46]. The tailored square resistance of the electrode is as low as 0.3 MΩ/□, and the resistance can stay stable after mechanical bending. Though the light absorption of monolayer graphene is only about 2.3%, the absorption for specific wavelengths can be tuned by light-matter interaction like surface plasmon resonance (SPR) [47,48].

The hexagonal boron nitride (h-BN) is an intrinsic insulative material with a structure similar to graphene. The wide indirect bandgap of 5.9 eV donates its outstanding absorption in the deep-ultraviolet regions [49,50]. He et al. created a large-scale flexible solar-blind ultraviolet photodetector based on 2D h-BN nanopaper [51]. The surface defects on nanosheets can lead to the formation of depletion layers at the interface after the nanosheets are stacked in nanopaper, significantly reducing the device’s dark current. A sudden increase in the carrier concentration under illumination narrows the width of the depletion region, resulting in superior photoresponse of the device. In addition, Badhulika et al. [49]. combined drop-casting the h-BN nanosheets with flexible copper to fabricate the Schottky junction ultraviolet photodetector. Internal photoemission occurs in the heterojunction region, which improves the quantum efficiency.

#### 2.2.2. Two-Dimensional Monoelemental Materials (Xenes)

Xenes, as an emerging monoelemental 2D materials family, including phosphorene, tellurene, silicene, germanene, antimonene, borophene and so on, have attracted much attention in photonic applications.

Black phosphorus (BP) is a rhombohedral layered 2D material with a wrinkled honeycomb structure, exhibiting an adjustive direct bandgap (0.3 to 2 eV) depending on thickness [35,52]. The carrier mobility is measured to be the highest 1000 cm^2^V^−1^s^−1^ in 10 nm thickness BP, superior to most 2D semiconductors [53,54]. In addition, BP demonstrates in-plane anisotropic optical absorption and electrical transportation properties, making it an excellent material for infrared polarization photodetection. Feng and co-author demonstrated a BP photodetector by delaminating BP crystals to few-layer BP nanosheets in an electrochemical manner, which shows a broadband photoresponse from 532 to 1850 nm [55]. A serious problem is that few-layer BP films are easy to oxidize and degrade in ambient conditions, restricting the research and application of BP. Thus, surface coating with materials such as Al_2_O_3_ or h-BN has been developed to physically isolate water and oxygen in the atmosphere, aiming to improve the stability of BP photodetectors [54,56]. Additionally, the use of chemical functionalization to modify surface sites on black phosphorus has also been employed to enhance its stability. For example, Sun et al. deposited 15 nm thickness pentacene on BP nanosheets, forming a passivation and N doping layer [57]. The device keeps a polarization photoresponse in a broadband region from visible to near-infrared, and the photoresponse remains equivalent to its initial level after two weeks.

Although theoretical predictions suggest that Xenes materials possess excellent optoelectronic properties, most research remains focused on black phosphorus. In addition to phosphorene, semiconductor Xenes such as tellurene [58], silicene [59], and antimonene [60], have also been reported for optoelectronic detection applications. Similar to black phosphorus, tellurene has a narrow bandgap. The experimentally measured carrier mobility is 700 cm^2^V^−1^s^−1^, enabling optoelectronic detection over an ultra-wide range (520 nm–3.39 μm) [58]. Moreover, the air stability of tellurene is superior to that of black phosphorus. Even when placed in air for two months, its electrical properties can still remain stable. These excellent properties make tellurene promising to replace black phosphorus or commercial InGaAs materials and show great potential in high-speed and high-performance infrared detectors. Although silicene belongs to Group IV elements like carbon, its honeycomb structure is not as flat as that of graphene but has an inherent buckling, resulting in the breaking of mirror symmetry. Theoretical predictions indicate that silicene has Dirac fermion properties similar to those of graphene and exhibits semi-metallic properties (with a bandgap of 1.55 meV), which somewhat limits its application in the optoelectronics field. However, through means such as defect engineering and surface modification, the bandgap of silicene can be tuned, thus expanding its application scope [61].

The research on Xenes materials is still in its infancy, and the exploration in the optoelectronics field is far from reaching the depth of graphene and transition metal dichalcogenides (TMDCs). With the development of more research, their potential application value will be more fully exploited.

#### 2.2.3. Transition Metal Dichalcogenides (TMDCs)

TMDCs are a group of 2D materials composed of transition metals and chalcogens, which can be described by the common chemical formula of AB_2_. A represents the transition metals such as Mo, W, and Re, and B represents the chalcogens such as S, Se, and Te [7]. TMDCs with semiconductor phase have layer-dependent bandgaps, which typically vary from 0.8–2 eV, making them suitable as channel materials for flexible photodetectors in the visual and near-infrared regions [62]. MoS_2_ is the most studied material in the TMDCs family. High carrier mobility, Young’s modulus, optical absorption, and ambient stability make MoS_2_ a prominent material in flexible photodetection applications [63,64]. Bo Sun et al. presented a flexible MoS_2_ photodetector enhanced by Ag nanocubes localized surface plasmon on PET film. The device enhanced by Ag nanocubes exhibits an ultrahigh responsivity of 7940 A/W and can maintain good mechanical endurance during 10,000 bending cycles [39]. Some TMDC semiconductors, such as ReS_2_ and ReSe_2_, demonstrate opposite unique distorted octahedral structures, endowing them with in-plane anisotropy in both optical and electrical properties. Liu et al. [65]. demonstrated that ReS_2_ crystals exhibit stronger absorption along the b-axis direction compared to the perpendicular direction, resulting in a linear dichroic photocurrent in an intrinsic ReS_2_ photodetector.

Among the TMDCs crystals, Dirac/Weyl semimetals, including WTe_2_, 1T’ MoTe_2_, PtTe_2_, etc., with gapless band structure have also garnered much attention since the broadband photoresponse covering the UV to the far-IR spectral regime [66,67,68]. In addition, the semimetal TMDCs have ultrahigh carrier mobility and can be used in high-frequency photodetection. The photocurrent anisotropy ratio can reach 2.25 at 1550 nm, and the photodetection range can extend to 10.6 μm in 1T’ MoTe_2_-based photodetectors, which makes them appealing for infrared polarization detection [69,70].

#### 2.2.4. Two-Dimensional Transition-Metal Carbides, Nitrides, and Carbonitrides (MXenes)

Mxenes, a large category of the 2D materials family, are produced by selectively removing certain atoms from the MAX phase precursors [71,72]. They possess a general stoichiometry of M_n+1_X_n_T_x_, where M is an early transition metal, X is carbon and nitrogen, and T stands for termination (such as -OH, -F, -Cl) absorbing in the etchant. Mxenes have many fascinating electrical, optical, and mechanical properties, making them promising for photodetection applications. Although many Mxenes show metallic characters (such as Ti_3_C_2_T_x_), they can play a role in charge transfer or constructing Schottky heterojunctions with other 2D materials. Graphene has been widely used as a transport material in photodetectors, but recent research utilized Ti_3_C_2_T_x_ as the transport layer in a paper-based ReS_2_ photodetector [73]. Compared with the graphene transport layer, the responsivity of the photodetector is promoted enormously in the Ti_3_C_2_T_x_ transport layer device, either in the visual region or the NIR region. Zeng et al. utilized the high conductivity and broad spectrum absorption in metallic Mxenes to fabricate a Ti_3_C_2_T_x_/GaAs Schottky heterojunction photodetector [74]. The device breaks the absorption edge of GaAs and expands the detection wavelength to 980 nm. On the other hand, Mxenes can also act as semiconductive sensitive materials by adjusting the functional group. Shen et al. modified the termination of Ti_3_C_2_T_x_ with dodecyl (-C_12_H_26_), fabricating a semiconductive Ti_3_C_2_T_x_-C_12_H_26_ photodetector [75]. Like TMDCs, the semiconductive Mxenes also have a tunable bandgap of 0.49–2.15 eV as layer numbers change.

In addition to layered 2D materials, emerging non-layered materials such as CdSe, CdS, and PdS, also show excellent optical properties when they exist in 2D form, making them very suitable as photoactive materials [76,77]. Due to their non-layered structure, they generally need to be synthesized through a bottom-up approach, and there are dangling bonds on the surface, so it is possible to synthesize nanoplatelets with core-shell to achieve better optical performance [78].

### 2.3. Figures of Merit

A set of key figures of merit for PDs is used to evaluate the performance of PDs formed by different materials, mechanisms, and processes. Those parameters include responsivity (*R*), external quantum efficiency (EQE), noise equivalent power (NEP), specific detectivity (D*), response speed, and 3 dB bandwidth. In this part, we will introduce those parameters briefly.

(1) *R* is the photocurrent generated from per effective unit incident power on the effective area and can be calculated by the ratio of photocurrent and incident power:

(1) $$R=\frac{I_{ill}-I_{dark}}{P_{in}A_{d}}$$

where the *I_ill_*, *I_dark_*, *P_in_*, and *A_d_* are the current under illumination, dark current, incident power density, and effective area, respectively.

(2) EQE is used to describe the capability that converts photons to carriers of PDs and is defined as the ratio between the number of photogenerated carriers to the number of incident photons:

(2) $$EQE=\frac{N_{carrier}}{N_{photon}}=\frac{I_{ph}/e}{P_{in}/hv}=\frac{Rhc}{e\lambda }$$

where *e* is the elementary charge, *h* is the Planck’s constant, *ν* is the frequency of incident light, *c* is the speed of incident light, and λ is the wavelength of incident light

(3) *NEP* is defined as the minimum light power that can make the PDs achieve signal-to-noise of 1 at 1 Hz bandwidth. The *NEP* can be calculated by:

(3) $$NEP=\frac{{\bar{i^{2}}}^{1/2}}{R}$$

where ${\bar{i^{2}}}^{1/2}$ is the mean-square noise current, which is composed of thermal noise, shot noise, and flicker noise and can be obtained from the frequency-dependent noise spectral density at 1 Hz bandwidth. *NEP* can represent the minimum illumination power of detectability.

(4) D* represents the ability of photodetectors to detect weak light signals, reflecting the response sensitivity of photodetectors to noise. and can be expressed as:

(4) $$D^{*}=\frac{\sqrt{A_{d}B}}{NEP}$$

where *B* is the electrical bandwidth of the noise measurement.

(5) Response Speed and 3 dB bandwidth. Response time describes the fast reaction ability of PDs to incident optical signals. The times from irradiation to form maximum photocurrent and from shutting light to decay to dark current are called rise time and decay time, respectively. In general, the rise time refers to the time interval during which photocurrent rises from 10% to 90% of the maximum value and the decay time is in reverse. The photogenerated electron–hole pairs generate/stop when irradiation begins/stops. The time that the carriers transit the channel and are collected by electrodes determines the response speed. The photoresponse tends to decrease as there is an increase in optical switching frequency. When the photoresponse decreases to 0.707 of its steady maximum value, the modulation frequency is defined as 3 dB bandwidth. Response time and 3 dB bandwidth are parameters used to evaluate the response speed in the time and frequency domain, respectively.

## 3. Solution-Processing and Printing Assemble for 2D Materials

Solution-processing of 2D materials involves a top-down process to exfoliate 2D materials bulks into stable few-layer nanosheet dispersions, including liquid-phase exfoliation (LPE) and electrochemical exfoliation (ECE). Solution-processing offers notable properties such as low cost, high performance, and easy processing, and has prominent superiority in turning laboratory-scale research into industrial applications. This section introduces the primary solution-processed method to exfoliate 2D materials into printed ink, including LPE and ECE. Then, we introduce the common printing technologies.

### 3.1. Liquid-Phase Exfoliation of 2D Materials

**Figure 1:**
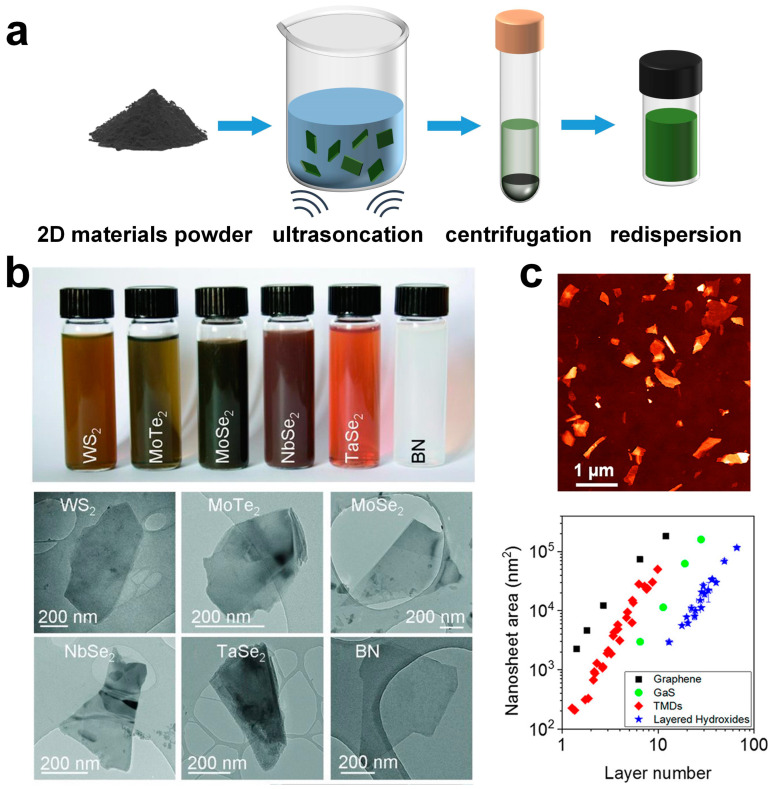
(**a**) Schematic of ultrasonication LPE. (**b**) The upper part is photography of WS_2_, MoTe_2_, MoSe_2_, NbSe_2_, TaSe_2_, and BN dispersions obtained by LPE (upper), and the lower part is TEM images of corresponding nanosheets in the upper [82]. (**c**) AFM images of graphite exfoliated in aqueous sodium cholate solution by tip sonication (upper) and plot of mean nanosheet area as a function of mean layer number [87].

LPE utilizes ultrasonication generating shear force from energy of the collapse of cavitation bubbles in specific solvents to exfoliate nanosheets from bulk crystal. In 2008, Coleman proposed, for the first time, exfoliated graphene and other 2D materials by ultrasonication in solvents [46]. The power and time of ultrasonication have a strong influence on the distribution of the thickness and lateral size of the exfoliated nanosheet. However, the lateral size of nanosheets varies from tens of nanometers to tens of micrometers, and the thickness is an average of less than 10 layers [79]. Figure 1a demonstrates the common process of LPE of 2D materials. Khan et al. presented a concentration and redispersion process to concentrate liquid-phase-exfoliated graphene as high as 63 mg/mL [80]. The dimensions of graphene are selected to ~1 μm in lateral length and ~3 layers in thickness. A variety of materials including graphene, BP, h-BN, TMDCs (e.g., MoS_2_, WS_2_, TaSe_2_, ReS_2_, InSe, and CrTe_3_), and Mxenes, have been exfoliated to nanosheets dispersions, with some examples shown in Figure 1b [51,52,81,82,83,84,85,86]. Prolonging ultrasonication power and time is feasible to improve yield, but the proportion of small fractions will increase. Films composed of low-aspect-ratio nanosheets have large contact resistance, affecting the photoelectric performance of the device [33]. In addition, even under the same ultrasonic conditions, there exists a wide range of nanosheet size distribution. The lateral size of exfoliated nanosheets exhibits a significant correlation with thickness (Figure 1c) [87]. Nanosheets with similar lateral sizes and thickness distributions can be obtained by adjusting the centrifugal acceleration.

It is important to note that the medium solvents are essential to exfoliate processing and dispersion stability. Based on Hansen’s solubility parameter theory, effective exfoliation occurs only when the surface energies between the solvent and the two-dimensional material are similar, allowing the exfoliated nanosheets to be stably dispersed through solvent–nanosheet interactions [88]. For instance, graphene has a surface energy of approximately 70 mJ/m^2^, and solvents such as N-methyl pyrrolidone (NMP) and N, N-dimethylformamide (DMF), which have surface energies of about 70 and 67 mJ/m^2^, respectively, are commonly utilized for the dispersion of graphene in solution-based processes. However, the organic solvents mentioned above that are effective in 2D materials exfoliation have high boiling points, which generally make the organic residue exist in the functional film based on the organic dispersion. The residue will hamper the performance of 2D materials. Thus, additional anneals are needed to remove residue completely. However, the high temperature during annealing is detrimental to flexible substrates. Driven by this consideration, low boiling point alternatives such as water, ethyl alcohol, and isopropanol are considered to serve as solvents in LPE. The mismatch in surface energy between the solvent and 2D materials results in poor exfoliation performance when using a single solvent. To ameliorate the problem, methods such as mixing the solution with another solution or with surfactants are adopted. Ajayan et al. developed a low-cost and low-toxic strategy of mixing water with common organic solvents (IPA, THF, and acetone) to match the surface energy of targeted 2D materials [89]. The binary cosolvents enable efficient exfoliation of many 2D material crystals, including graphene, MoS_2_, h-BN, and so on. Varrla et al. added natural Sapindus mukorossi (SM) surfactant into the water to stabilize graphene nanosheets during LEP [90]. The hydrophobic groups in SM absorb on the surface graphene, and the dissociated hydrophilic groups produce static repulsive forces against aggregation, which allow the graphene concentration to achieve 1 mg/mL in the surfactant-assisted aqueous within 1 h of ultrasonication.

Nevertheless, the ordinary yield of LEP is as low as several percent, which is still less than 20% after optimized processes. High-shear mixing during the exfoliation shows superior mass-production potential with a higher yield of 78.3%, which is an appealing industry-level production [91].

### 3.2. Electrochemical Exfoliation of 2D Materials

**Figure 2:**
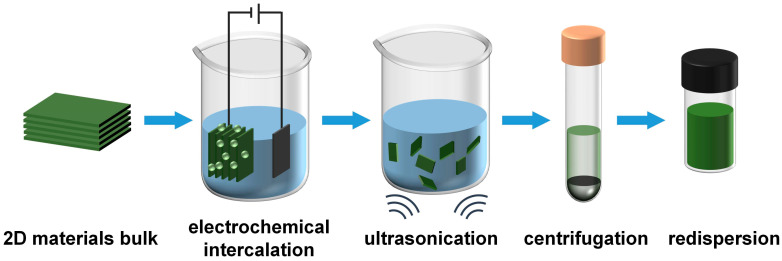
Schematic of electrochemically exfoliating 2D materials.

ECE is an emerging strategy to obtain 2D materials nanosheets. During this process, the working electrode (layered materials) and auxiliary electrode (e.g., platinum, graphite) are immersed into an electrolyte, as shown in Figure 2. An external direct current potential bias is applied to generate an electric-field force in conductive media, which drives the intercalation ions inserted into the van der Waals gaps of 2D materials. The volume of 2D materials bulk can expand several times after sufficient intercalation. Subsequently, the intercalated bulk can be exfoliated into high-quality and free-standing nanosheet dispersion by mild ultrasonication for a few minutes to hours.

**Figure 3:**
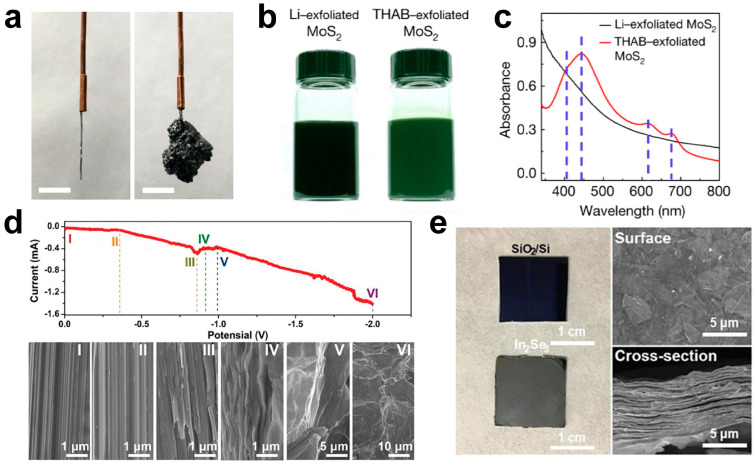
(**a**) The comparison of the MoS_2_ bulk crystal photographs before and after electrochemical intercalation by THAB. (**b**) Photographs of electrochemically exfoliated MoS_2_ nanosheets dispersion by Li^+^ ions and THA^+^ ions, respectively. (**c**) Absorption spectra of Li-exfoliated MoS_2_ and THAB-exfoliated MoS_2_. The blue dashed lines indicate the absorption peaks of THAB-exfoliated MoS_2_ [96]. (**d**) Linear sweep voltammetry of In_2_Se_3_ crystal electrode in THA^+^ electrolyte to identify the reaction stages (I–VI) of the intercalation process (upper) and SEM images of the In_2_Se_3_ cathode deformation at various intercalation periods (lower). (**e**) Photographs of the SiO_2_/Si and In_2_Se_3_ thin film on SiO_2_/Si (left) and top-down and cross-sectional SEM images of the In_2_Se_3_ film deposited on the substrate (right) [97].

Depending on the voltage applied to the bulk materials, the exfoliation can be divided into anodic exfoliation and cathodic exfoliation categories. In the anodic exfoliation process, the 2D materials are applied a positive voltage and act as the anodic of electrolytic tank [11,92]. Under the influence of the electric field, the anions in the electrolyte (such as Cl^−^, NO_3_^−^, SO_4_^2−^) are embedded into the bulk materials and undergo oxidation reactions, which generate gas in interlayer gaps and expand the crystal. Lee and co-workers obtained large-area MoS_2_ nanosheets with a lateral size of 50 μm by exfoliating MoS_2_ crystal with Na_2_SO_4_ solution [93]. However, the oxidation of nanosheets tends to occur together with the intercalation of anions. During cathodic exfoliation, a negative potential is applied to the working electrode. Inorganic alkali metal cations (such as Li^+^, Na^+^, and K^+^) are firstly preferred to employ as intercalation ions because of their smaller radius [92,94]. Though high yield, the guest intercalation of alkali metal might induce undesired phase transition to some host crystal, which can change the phase of exfoliated nanosheets from semiconductor 2H to metal 1T and make it unsuitable for photodetection application [16,88,94,95]. Duan et al. proposed a quaternary ammonium cation (tetraheptylammonium bromide, THAB) intercalated method to prevent the electrons from injecting into crystals (Figure 3a–c) [96]. The exfoliated MoS_2_ nanosheets have a narrow thickness distribution, a pure 2H semiconductor phase, and an extremely high yield of 95%. Feng et al. utilized linear sweep voltammetry to analyze the intercalation process of THA^+^ into In_2_Se_3_ (Figure 3d,e), finding that the THA^+^ can insert into In_2_Se_3_ host crystal and combine with In_2_Se_3_ to (THA^+^)*_x_*In_2_Se_3_*^x^*^−^, then subsequently decompose to trihexylamine and alkanes [97]. Defect-free In_2_Se_3_ nanosheets with a high yield of up to 83% are feasible for printing photoelectronic sensors in the wearable applications.

### 3.3. Printed Flexible Photodetector: Technology and Performance

It is most important to scalably assemble solution-processed 2D material nanosheets into continuous channels in the fabrication of photodetectors. A high-quality dispersion of 2D materials obtained by solution processing is natively suitable to act as the ink of various printing techniques. In general, the performance of printed photodetectors is determined by the quality of the ink and the printing method used. In this section, the different assembly methods in the category of printing are discussed, including spray coating, screen printing, inkjet printing, electrohydrodynamic jet printing, and aerosol jet printing. The properties of all the printing techniques are compared and tabulated in Table 1.

#### 3.3.1. Spray Coating

**Figure 4:**
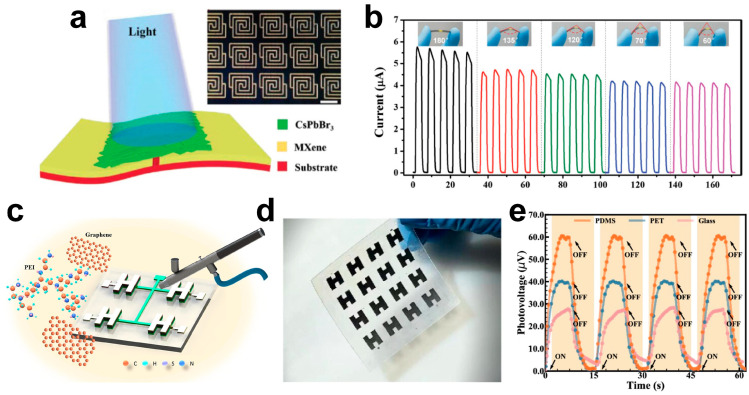
(**a**) Schematic of the CsPbBr3 photodetector with MXene electrodes, insert is the part image of MXene electrodes array. (**b**) Stability of the flexible device under the different bending angles [15]. (**c**) Schematic of the spray coating process of graphene/polyethylenimine composite photothermoelectric detector. (**d**) Photograph of 16 H-shape cells of graphene/PEI detectors. (**e**) I–t curves of graphene/polyethylenimine detectors under 973 K radiation with PDMS, PET, and glass substrates [18].

The ink is atomized into small aerosol drops in spray coating through ultrasonic vibrations or gas pressure. The droplets are carried out of the nozzle by a carrier gas (ordinary inert gas) and sprayed onto the substrate randomly [16,17]. The sprayed drops do not possess the ability for directional deposition. Therefore, it is necessary to place corresponding masks on the substrate for precise positioning when depositing films with specific patterns. The thickness of spray-coated films can be adjusted by controlling the moving speed of the nozzle and the flow rate of the solution. For films that require patterning, spray coating may lead to a waste of ink. As shown in Figure 4a, an all-sprayed flexible photodetector array with 1665 pixels in 72 cm^2^ based on 2D perovskite/MXene nanosheets was demonstrated for photo-communication, showing an outstanding photo-to-dark current ratio of 2.3 × 10^3^ at 450 nm [15]. The device exhibits good stress relief capability and the photoresponse decreases slightly after the bending angle reaches 135° (Figure 4b). However, the D* is just 6.4 × 10^8^ Jones and responsivity is 44.9 mA/W under 10 V bias. Recently, Xie et al. reported a composited graphene/polyethylenimine flexible photothermoeletric detector by spray-coating method to realize mid-infrared blackbody radiation detection (Figure 4c–e), achieving a photovoltage *R* and D* of 2.7 V/W and 6.05 × 10^7^ cmHz^1/2^W^−1^, respectively [18]. The spray-coated photodetector realizes a controllable, scalable fabrication process. Besides graphene, TMDCs, including MoS_2_ and MoSe_2_, have also been applied to spraying coated photodetectors. Lobo et al. sprayed liquid-phase-exfoliated MoS_2_ nanosheet dispersion onto screen-printed carbon electrodes, fabricating a paper-based flexible photodetector [19]. The device was calendared under rollers to enhance the performance by reducing the voids in the paper and scattering points in inter-nanosheet junctions, showing a *R* of 6 mA/W at an illumination density of 2.5 mW/cm^2^. Although the minimum line width achieved through spray coating is typically greater than 1 mm, which restricts the photodetection performance of devices, this rapid and low-cost fabrication method provides a viable technological pathway for the large-scale flexible integration of two-dimensional materials.

#### 3.3.2. Screen Printing

**Figure 5:**
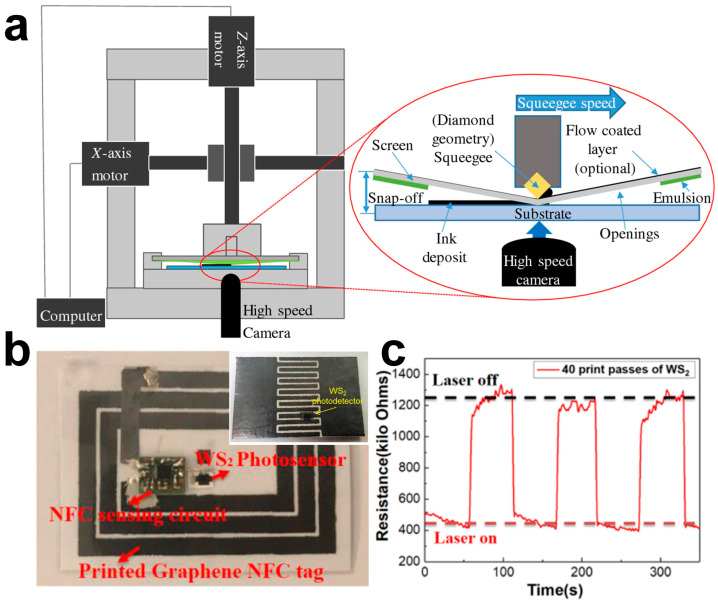
(**a**) Schematic diagram of the screen-printing device (left) and the zoomed schematic of the screen printing process (right) [20]. (**b**) Image of inkjet-printed WS_2_ photodetector integrated with screen-printed graphene NFC tag. The insert is an image of an inkjet-printed WS_2_ photodetector with a screen-printed graphene meandered line. (**c**) The transit response of WS_2_ photodetector with 40 print passes recorded by wireless NFC tag [21].

Screen printing is a widely implemented technique in garment manufacturing that involves using a stenciled mesh to transfer the ink to target substrates in accordance with the designed pattern [7,17]. As shown in Figure 5a, a squeegee pushed the ink from one side of the stenciled mesh to the other [20]. During the process, the mesh is brought into contact with the substrate, and the ink passes through the apertures of the mesh to complete the printing. Compared with other printing methods discussed in this review, the major features of screening printing are higher ink viscosity, convenient patterning operations, and contact with the substrate. As shown in Figure 5b,c, screen-printed graphene has been reported to act as source and drain electrodes and near-field communication tag antenna in a wireless WS_2_ photodetector [21].

#### 3.3.3. Inkjet Printing

Compared with spraying coating and screen printing, inkjet printing is a versatile and cost-effective technique that can deposit 2D materials ink as specific pattern with high accuracy [7,17,99]. Features such as being non-contact, maskless patterning, low waste, and scalability to large-area fabrication make inkjet printing promising for optoelectronic devices.

**Figure 6:**
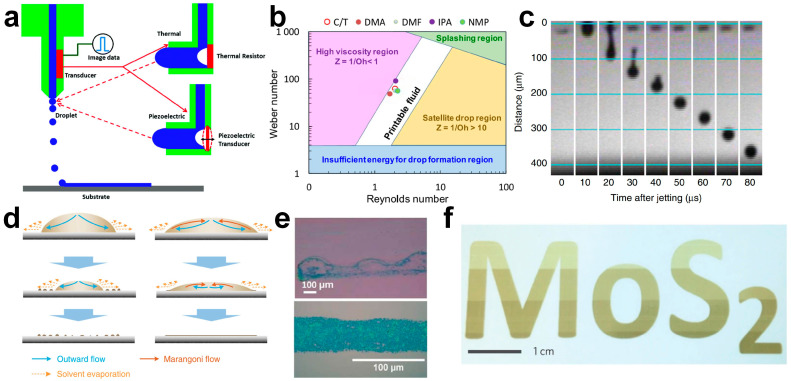
(**a**) Schematic illustration of drop on demand inkjet printing [28]. (**b**) Illustration of inkjet performance effected by ink fluidic property [105]. (**c**) Droplet jetting sequence observed from the printer stroboscopic camera. (**d**) The comparation of droplet drying process without (left) and with (right) a recirculating Marangoni flow induced [35]. (**e**) Optical pictures of inkjet-printed graphene line with and without affect by coffee ring effect. (**f**) Printed ‘MoS_2_’ with water-based MoS_2_ ink on PEL P60 paper with an increasing number of printed passes (from 1 to 4, top to bottom) [22].

The schematic diagram of inkjet printing is shown in Figure 6a. Two jetting modes are available in inkjet printing: continuous inkjet (CIJ) and drop-on-demand inkjet (DoD) [28,100,101]. In a CIJ system, the chamber pressurizes at a specific pressure, and the ink ejects from the nozzle in a stream of droplets. The droplets are selectively charged during the generation process and the charged droplets are separated by a deflection electric field to deposit on the substrate. In addition, the uncharged droplets are carried back to the ink chamber to avoid wasting. Although CIJ printing can achieve high-frequency jetting and high-speed printing, its lower resolution and higher system complexity make DoD more preferred [28,102]. In a DoD system, inkjet printing squeezes the ink in a drop-by-drop manner. The ink was divided into chambers and squeezed by a piezoelectric nozzle from the nozzle. The chamber volume is contracted under an external voltage on account of the piezoelectric effect. This sudden reduction generates an impact force in the liquid, which overcomes the surface tension stress in the liquid surface and squeezes the drop from the nozzle. During the whole process of inkjet printing, droplets are generated from the piezoelectric nozzle, ejected onto the specific location of the substrate as the movement of the nozzle, and ultimately evaporate the solvents to form a film. The forming of droplets in printing is mainly determined by nozzle diameter and physical properties of ink, among which surface tension γ, viscosity η, and density ρ are the most critical parameters [25,103]. During printing under DOD mode, a single droplet may be followed by satellite or secondary droplets, which tends to reduce the resolution in the printing process [104]. Fromm proposed a dimensionless figure Z, which is the inverse of Ohnesorge number, =(Oh)^−1^ = (γρα)^1/2^/η to evaluate the quality of ink to obtain a better print effect. If Z is in a suitable range, there are few satellite droplets around printing routes. Derby and Reis researched a simple, single-orifice, piezoelectric inkjet droplet generator and proposed that inkjet printing is conducted in the range of 1 < Z < 10 (Figure 6b,c) [101,105]. If Z is small, the ink is ropy, and the fluid column extension following the main droplet is short. However, the nozzle is easy to clog if the viscosity is high, and this value is usually advised to restrict below 35 cps [30]. On the opposite, the fluid column extension behind the droplet is significant if the Z of the ink is high, which leads to forming a satellite drop behind the main drop. Therefore, the viscosity and pressure pulse are important factors to the minimum of Z, which determine whether the droplets can be extruded, while the minimized forming satellite droplets determines the upper limit of Z. Notably, even in the case that Z > 10, stable jetting can still be achieved through optimizing electrical parameter [35,104].

Once ejected and deposited, the evaporation behavior of the droplets is also affected by the surface energy between the ink and the substrate interface, which can be calculated by measuring the contact angle between the ink and the substrate [105]. As the solvent evaporates, solids in ink remain on the substrate in a ring shape along the perimeter, just like a coffee ring [104,106]. The coffee ring effect would affect the homogeneity of the deposited film. The liquid at the periphery evaporates faster than in the center area, which causes the liquid in the center to flow outwards and replenish the edge, driven by the surface tension gradient, forming an outward convective flow. The drying edge pin on the contact edge causes the edge to be thicker than the center in the dried pattern [106]. This phenomenon can be suppressed by adopting a dual solvents strategy. A component with a high boiling point and surface tension is called a major solvent, and another with a lower boiling point and surface tension [107]. The dual solvents system can adjust the physical parameters (γ, ρ, η) of the ink. The difference in boiling points between the two components results in different evaporation rates. The solvent on the periphery evaporates faster than in the center. Thus, the more volatile solvent possesses a higher proportion in the center, which causes the surface tension gradient and induces an inward Marangoni flow (Figure 6d) [7,35,107]. The opposite convective flow and Marangoni flow continuously drive the circulation of particles in drop, producing a uniform assembly of the ink content [28]. Hu et al. disperse BP in IPA solvent and add 10 vol% secondary alcohol (2-butanol) to formulate the BP ink [35]. The ink droplets form a recirculate Marangoni flow to prevent coffee ring formation. Hersam et al. utilize a mixture system of cyclohexanone/terpineol with a volume of 85:15 to disperse MoS_2_ nanosheets to optimize the ink formation and minimize coffee ring effects [24].

**Figure 7:**
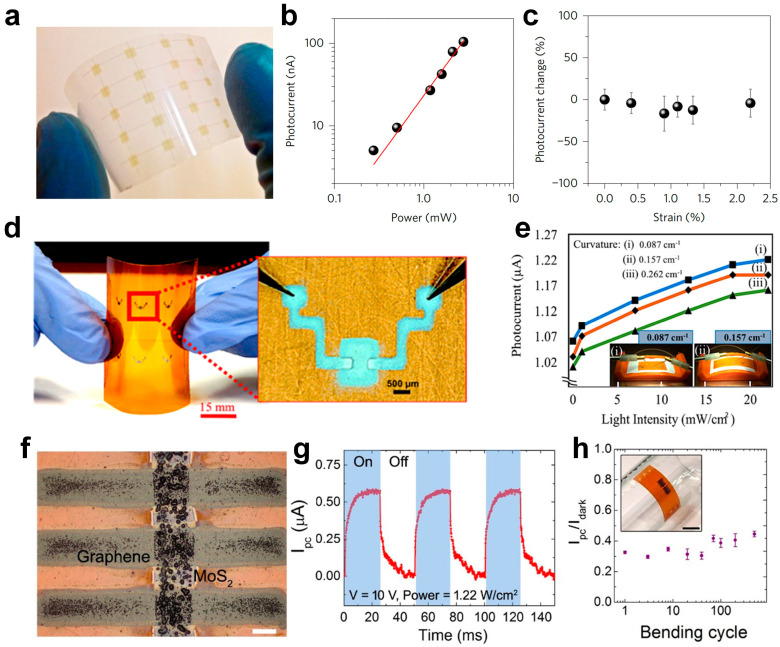
(**a**) Photograph of all inkjet-printed graphene/WS_2_/graphene photodetector on PET substrate. (**b**) Photocurrent as a function of incident power under 514 nm illumination. (**c**) Bending test of all-printed heterojunction [22]. (**d**) Photograph of all-printed graphene/MoS_2_/graphene array on 6 × 5 cm PI substrate. (**e**) Photocurrent as a function of light power density at different curvatures [23]. (**f**) Optical microscopy photography of inkjet-printed MoS_2_ photodetector with graphene electrodes on the PI substrate. (**g**) The photoresponse of photonic annealed PD in (**f**). (**h**) Bending test over 500 cycles showing invariant sensitivity. Inset: photograph of the flexible MoS_2_-Gr device. The scale bar is 3 mm [24].

Various flexible 2D materials photodetectors have already been realized through the inkjet printing process. One of the first fully inkjet-printed 2D material flexible PDs was fabricated by printing graphene/WS_2_/graphene heterojunction onto PET film and paper (Figure 7a–c) [22]. The photocurrent of device on PET substrate is unsaturated even at 3 mW illumination and stable under 2% strain. The printed paper-based device exists a *R* higher than 1 mA/W at 1 V voltage and laser power of 7 mW (λ = 514 nm). Dose-escalation cytotoxicity assays of cells prove that the water-based ink formulation modified by xanthan gum is biocompatible, which extends the potential applications to biomedicine. Although the inkjet-printed graphene/WS_2_/graphene array only comprises 16 detection units, the process of directly printing 2D material ink onto a flexible substrate has provided inspiration for the fabrication of all-2D flexible PD arrays and holds promise for large-scale roll-to-roll production. At the same time, Hossain and coworkers proposed a biocompatible and all-printed MoS_2_/graphene photodetector on PI substrates, as shown in Figure 7d,e [23]. The device shows a broadband photoresponse in the visible regime and exhibits a high *R* of 0.3 A/W and D* of 3.6 × 10^10^ Jones. The non-toxicity and stability make it suitable for the photoreceptor layer in retinal prosthesis. Similarly, Seo et al. fabricated a fully inkjet-printed flexible MoS_2_ photodetector with graphene electrodes on PI substrates (Figure 7f–h) [24]. Liquid-phase-exfoliated 2D materials nanosheets were dispersed into a dual solvent system of cyclohexanone/terpineol for inkjet printing to relieve coffee ring effects. The authors adopted photonically annealing to remove the polymer stabilizer and enhance electrical contact between the nanosheets, providing a responsivity of 50 mA/W and detectivity of 3.18 × 10^9^ Jones at 515 nm laser irradiation. Inkjet printing has a higher resolution (typically 20–50 µm) than spraying or screen printing, so devices prepared by inkjet generally have better optoelectronic detection performance. In general, the printed 2D materials films required annealing to improve the interface contact. The utilization of photonic annealing offers the flexibility to implement different annealing strategies for different material regions, allowing separate treatment for sensitive regions and electrode regions. This capability provides a flexible option for modifying the performance of inkjet-printed photonic devices.

**Figure 8:**
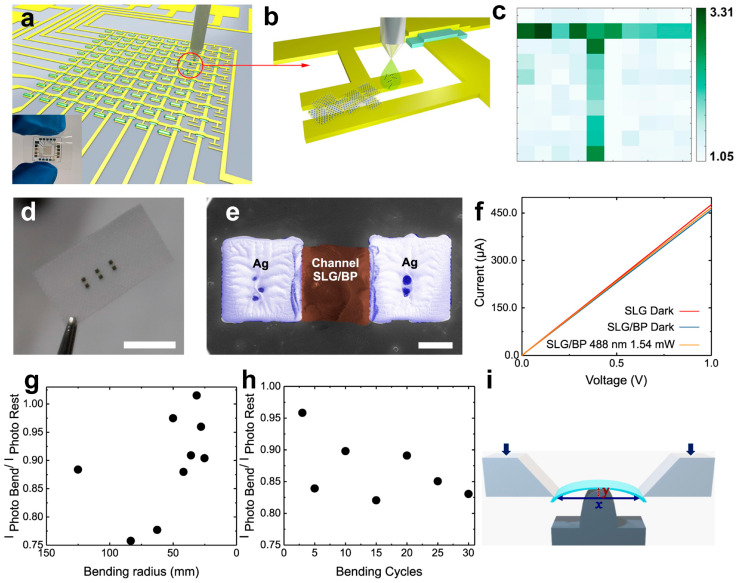
(**a**) Schematic of processing of printing flexible photodetector array. The insert is a photograph of the flexible photodetector array on a PET substrate. (**b**) Partial magnification of the printing process of a single unit of photodetector array. (**c**) The pixel graph of the “T” mask collected by the photodetector array [25]. (**d**) SLG/BP PD array on polyester fabric. Scale bar 5 mm. (**e**) SEM of PD on polyester fabric. Scale bar 200 µm. (**f**) Current as a function of *V* for SLG and SLG/BP in the dark, and SLG/BP under illumination at 488 nm. (**g**) Normalized *I*_photo_ as a function of bending radius. (**h**) Normalized *I*_photo_ as a function of the bending cycle. (**i**) Schematic of bending setup. Arrows indicate applied force on grips, the bent SLG/BP PD is drawn in mint green between the grips, y is the height at the chord midpoint, and x is the chord circumference [27].

Although the devices mentioned above are all fully inkjet-printed flexible 2D material PDs, there is a significant gap in terms of both photodetection performance and pixel density required for imaging applications. The development of PD array towards high-density integration is inevitable in the realization of photoimaging. In this aspect, Sun et al. proposed an ECE coupled with an inkjet printing strategy to fabricate a flexible photodetector array [25]. The exfoliated MoS_2_ ink is filtered through a syringe filter to obtain nanosheets with appropriate sizes, thereby preventing nozzle clogging during printing. As shown in Figure 8a,b, they integrated 100 PD units in a 9 mm^2^ area through DoD inkjet printing mode. The flexible photodetector array can reach a maximum D* of 1.82 × 10^11^ Jones under 520 nm illumination and maintain 80% photocurrent after bending at 10 mm curvature for 3000 times, which guarantees the clear photoimaging for recognizing the letter “T” in Figure 8c. Wang and cooperators prepared water-based 2D materials ink of MoTe_2_, MoS_2_, and graphene and fabricated MoTe_2_/graphene and MoS_2_/graphene heterojunction photodetectors on flexible PI substrate [26]. The inkjet-printed MoTe_2_ nanosheets show both semiconducting 2H phase and metallic 1T’ phase, including 120 mA/W responsivity under 532 m and 7.7 mA/W under 940 nm. Ferrari et al. fabricate flexible BP/graphene photodetector on textile fabric through a commercial inkjet printer for the first time (Figure 8d–f) [27]. The BP nanosheets obtained by LPE are dispersed in low-boiling-point and non-toxic IPA solvent without any other additive. Although the calculated Z value is 35, far beyond the conventional optimal range, they also realize stable inkjet printing. The performance of the device is comparable to others 2D material-based devices. But the *R* of the SLG/BP PDs on the textile fabric is only 6 mA/W at 488 nm, much lower than that on the SiO2/Si substrate; this is due to the fact that both the electric field at the SLG/BP interface the carrier mobility of SLG are greatly reduced on the fabric. As shown in Figure 8g–i, when the bending radius changes from flat to 25 mm, or after 30 cycles of bending at a radius of 35 mm, the device’s performance decreases by no more than 20%.

#### 3.3.4. Electrohydrodynamic (EHD) Jet Printing

**Figure 9:**
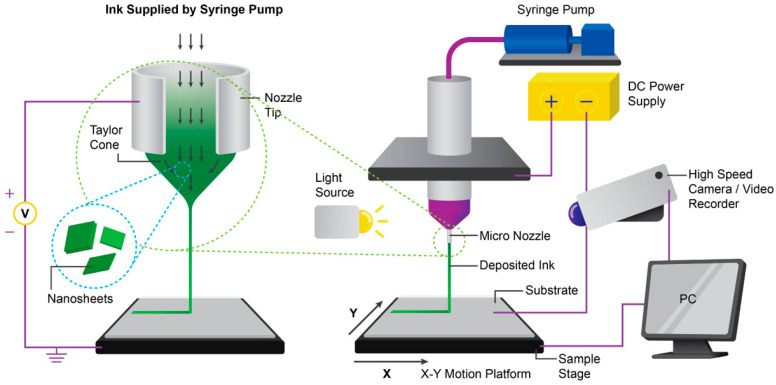
Experimental setup for the EHD printing technique [31].

The peculiar characteristics of EHD jet printing include higher printing resolution (<1 µm) and wider material compatibility (viscosity 1–10,000 cps) compared with ordinary inkjet printing. EHD jet printing can offer a minimum line width to the nanoscale, providing an attractive method for high-density photodetector integration. Figure 9 shows the schematic diagram of EHD jet printing. EHD jet printing needs a strong electric field between the nozzle tip and the substrate to create electrostatic “pulling” on the liquid surface, which makes EHD jet printing print high-viscosity inks without worry about nozzle blockage. When the electrostatic force overcomes the surface tension, liquid forms a microjet and jet from the nozzle in the form of a Taylor cone [108]. In particular, EHD jet printing can realize high-resolution deposition by adjusting the Taylor cone, and the line width can reach tens of nanometers. In addition, it can realize controllable jetting, just as inkjet printing, for specific patterns, which is a transformative additive nanomanufacturing to replace traditional photolithography in flexible device manufacturing. The complex deposition and patterning of ink droplets are achieved in combination with the movement of the substrate stage (X_Y direction) and nozzle (Z direction).

**Figure 10:**
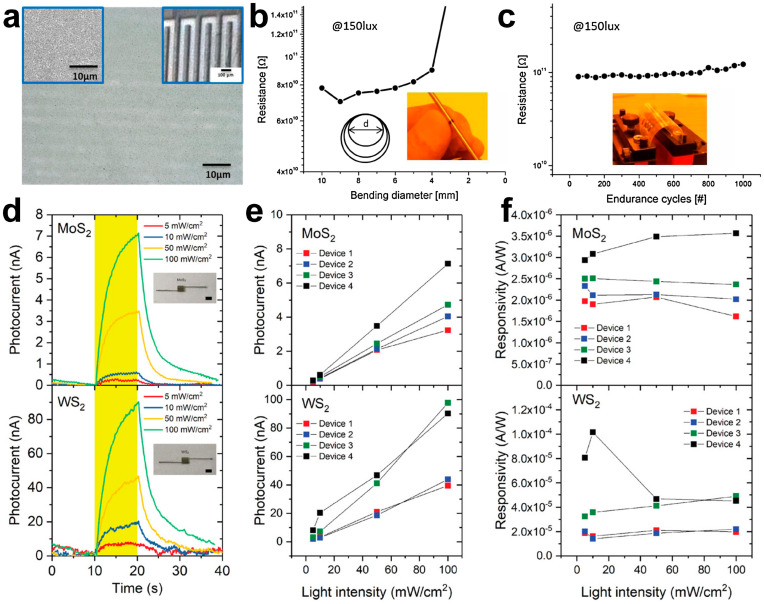
(**a**) Microscope image of the photo conductive film (scale bar 10 μm), where the top right inset shows the zoomed image of the comb type electrodes (scale bar 100 μm) and left top inset shows zoomed image of the silver electrode (scale bar 10 μm). (**b**) Bending analysis of the photo sensor device using metallic rod (10–0 mm) diameter. (**c**) Endurance cycle analysis of the fabricated device by bending 1000 times at 4 mm [29]. (**d**) Photoresponse of MoS2 and WS2 photodetectors under different incident power. (**e**) Photocurrent (total current–dark current) of four similar MoS_2_ and WS_2_ photodetectors. All four devices show comparable photocurrents in response to the illumination. (**f**) Responsivities of the photodetectors shown in (**d**) [31].

As shown in Figure 10a, Ali and colleagues proposed a graphene-based composite photodetector through EHD [29]. The sensing layer (120 nm perylene/graphene composite thin film) and silver electrodes (350 nm) are sequentially deposited on PET substrate through EHD jet printing. The electrodes were designed to comb type and, thus, improved sensor sensitivity. The response of the device is concentrated in the visible range and exhibits normal behavior for bendability from 10 mm to 4 mm (Figure 10b). Furthermore, it demonstrates stable performance when the device is bent around a 4 mm diameter rod up to 1000 times, as shown in Figure 10c. Similarly, an in situ deposited graphene photodetector was printed on a polymer substrate through drop on demand mode of EHD jet printing [30]. Alzakia et al. exploited an ultrafast LPE technique and fabricated all-2D-materials MoS_2_ and WS_2_ photodetectors with graphene electrodes through one-step EHD jet printing (Figure 10d–f) [31]. After 6 h sonication, the exfoliation yield increases more than 100 times, reaching the desired level required for printing applications, above 1 mg/mL. The photocurrent of MoS_2_ PD and WS_2_ PD can reach the order of 10^−9^ A and 10^−7^ A, but due to the large channel size (~2 mm), the *R* are all at μA/W level. Anticipating printed 2D material ink directly, Hong et al. proposed an economical, rapid, scalable solution for high-performance MoS_2_ photodetectors by EHD-jet printing ammonium tetrathiomolybdate precursor solution [32]. The precursor can convert to four to five layers thickness MoS_2_ after the pre-annealing and annealing process. The photodetector has a high photosensitivity of 3444% and a photoresponsivity of 1.68 AW^–1^.

#### 3.3.5. Aerosol Jet Printing (AJP)

Aerosol jet printing is an emerging direct additive printing technology that has been used in the fabrication of flexible electronic devices. In this process, the atomization gas flow carries ink through an atomizer to form an aerosol stream and transport the aerosol stream to the deposition head, in which the aerosol is focused onto the substrate by sheath gas flow [109,110]. Depending on the atomize mechanisms, the AJP can be divided into pneumatic APJ and ultrasonic APJ.

**Figure 11:**
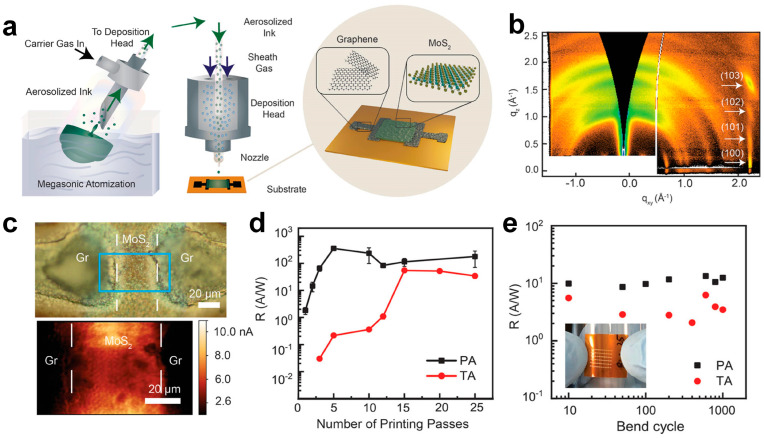
(**a**) Schematic showing AJP deposition and printed photodetector geometry. (**b**) Grazing-incidence wide-angle X-ray scattering map of AJP printed MoS_2_ film. (**c**) Optical microscopy image of a PA device. The corresponding spatially resolved SPCM map of the scanned area, measured at an applied bias of 4 V with a 630 nm laser at a power of 41 μW. (**d**) Responsivity as a function of the number of printing passes. (**e**) Bending stability over 10^3^ cycles with a bending radius of 12 mm [33].

The work by Hersam et al. demonstrated a micrometer scale controlled all-2D materials flexible photodetector by megasonic atomization AJP [33]. Figure 11a represents the process illustration of aerosol jet printing MoS_2_/graphene device, where the photoactive MoS_2_ nanosheet network and graphene electrodes are well-aligned on the polyimide substrate. Grazing-incidence wide-angle X-ray scattering mapping in Figure 11b shows uniaxial texture along the *c*-axis of the MoS_2_ film. Photonic annealing forms quasi-ohmic contact between MoS_2_ and graphene (Figure 11c–e), which guarantees a high responsivity of 10^3^ A/W, a fast response speed of about 1 ms, specific detectivity of 1.8 × 10^7^ Jones, and stable photoresponse after 1000 bending cycles under 515.6 nm irradiation, superior to thermal annealing.

**Figure 12:**
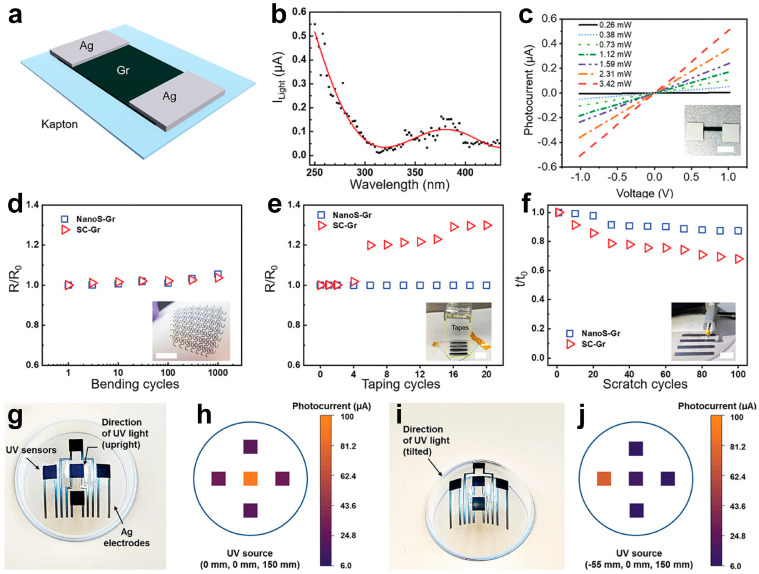
(**a**) Schematic illustrations of the in-plane device of Ag/NanoS–Gr/Ag. (**b**) The photocurrent of NanoS–Gr over different excitation wavelengths of light. (**c**) The I–V curves corresponding to the photodetector shown in (**a**) under UV illumination. (**d**) Relative resistance (*R*/*R*_0_) measured as a function of bending cycles for graphene films printed on polyimide substrates (bending radius of 12 mm). The inset image shows a printed serpentine pattern (scale bar: 1 cm), indicating the potential of fabricating flexible/stretchable devices. (**e**) Resistance of graphene films versus number of tape pulling. Inset shows a graphene film under taping test with a 10 mm scale bar. (**f**) Durability test of printed graphene films by measuring the film thickness change during 100 scratch cycles. The inset shows a NanoS–Gr film under scratching test with a tip radius of 2 µm and a scratch force of 10 mN. Scale bar in the inset is 5 mm. (**g**,**i**) photographs of AJP conformal 3D PD array. (**h**,**j**) photocurrent mapping of light irradiation from directions in (**g**), (**i**), respectively [34].

AJP offers greater flexibility in substrate selection than other printing technologies. It does not require an external electric field as the ink droplets are driven by gas, and the distance between the nozzle and the substrate can be adjusted flexibly from 1–11 mm. This flexibility makes aerosol jet printing advantageous in manufacturing flexible, curved, and conformal devices. Zhang et al. printed a water-based nanosurfactant (NanoS) decorated graphene pattern on polyimide substrates (Figure 12a) [34]. The graphene device doped with NanoS exhibits a photoresponse peak at 385 nm (Figure 12b). As shown in Figure 12c, the all-printed lateral NanoS–Gr UV photodetector with silver electrodes exhibits linear and symmetric I–V characteristics, indicating a good Ohmic contact. The printed graphene electrode shows good structural stability and the change in relative resistance is negligible after 1000 bending cycles (bending radius of 12 mm), 100 taping cycles, and 100 scratching cycles (Figure 12d–f). The device shows photocurrent density of 2.6 μA/cm^2^ under 3.42 mW incident light irradiation. Furthermore, the functional materials are deposited directly on 3D glass and PDMS hemisphere with complex five sensing architectures, which facilitate the realization of next-generation flexible compound eye mimicking image sensors. This work demonstrates the significant potential of AJP to replace traditional lithographic processes (vacuum sputtering, lithographic patterning, and acidic etching) and to achieve 3D conformal deposition of functional materials on complex geometric surfaces for flexible applications.

## 4. Summary and Outlook

This review summarizes the recent progress of various flexible 2D materials photodetectors fabricated using printed 2D materials nanosheets. The native layered structure of 2D materials facilitates the solution-processing of exfoliating nanosheets into functional ink. Coupling exfoliated 2D materials ink with printing techniques will bring a revolution in device fabrication, functions, and layouts, replacing CVD in flexible photodetectors. Printing techniques are low-cost, highly effective, and large-scale fabrication processes for flexible 2D material PDs. Two-dimensional materials in ink form are convenient for materials composition, doping, and for use in hybrids, which may pave new paths in exploring novel and superior devices with diverse applications. Other dimensional materials, such as perovskites, quantum dots, and nanowires, can also be synthesized using solution-based methods. These materials can be integrated with 2D materials through printing techniques to form mixed-dimensional heterojunctions, opening new avenues for developing devices with novel structures and optoelectronic properties. For instance, 0D/1D heterojunctions composed of quantum dots and 2D materials can simultaneously retain the high carrier mobility of 2D materials and the strong light absorption of quantum dots, enabling detectors to achieve both fast response times and high responsivity. Furthermore, the printing process can be seamlessly integrated with existing semiconductor processes to achieve hybrid integration of 2D and 3D materials on substrates like silicon (Si), germanium (Ge), and gallium nitride (GaN). This promotes the low-cost commercialization of high-speed, high-sensitivity optoelectronic detection devices.

We have discussed various 2D material nanosheets ink synthesis methods and printing processes. Despite the fact that it is promising to achieve the large-scale manufacturing of flexible 2D material PDs through printing functional ink, challenges are still crucial. For example, the performance of printed devices often falls short of expectations. Balancing the convenience of printing while maintaining the performance of 2D material flexible PDs is a key research focus. So far, it remains a significant challenge to stably deposit 2D material nanosheets over large areas with micrometer or sub-micrometer scale resolution through printing, which expresses higher requirements for hardware design and software control in the entire printing system. Apart from optimizing the printing process, some post-processing affects the optical and electrical properties of printed PDs. Channels in PDs are composed of tiny nanosheets. The contact between nanosheets and the adhesion of nanosheets to the substrate can significantly affect the performance of printed flexible PDs in durability and reliability. In addition, nanosheets in solution-processible ink inevitably have defects such as vacancies. The methods for obtaining defect-free 2D material nanosheets ink, as well as the techniques for curing defects in nanosheets through post-processing, still require further exploration.

Consumer electronics, Internet of Things, and healthcare are important application areas where the fabrication of low-cost and large-scale flexible photodetectors is highly appealing, providing a strong motivation for adopting printing manufacturing techniques. Although the printing techniques still have a way to go, we expect that with refinement in coupling solution-processible ink formation with printing processes, the printing techniques will trigger an unprecedented revolution to flexible 2D PDs.

## Figures and Tables

**Table 1:** The comparation of various printing techniques and their main characteristic parameters [7,17,98].

Printing Technology	Viscosity (cp)	Thickness (µm)	Patternable	Resolution (µm)	Speed	Contact
Spray coating	1–1000	4–10	Mask	1000	Fast	No
Screen printing	1000–10,000	0.1–25	Mask	50–100	Fast	Yes
Inkjet printing	1–20	<10	Digital	20–50	Slow	No
Electrohydrodynamic jet printing	1–1000	0.001–0.3	Digital	Less than 0.2	Slow	No
Aerosol jet Printing	1–20	<10	Digital	20–40	Slow	No
